# Ultrastructural analysis of the dehydrated tardigrade *Hypsibius exemplaris* unveils an anhydrobiotic-specific architecture

**DOI:** 10.1038/s41598-020-61165-1

**Published:** 2020-03-09

**Authors:** Myriam Richaud, Emilie Le Goff, Chantal Cazevielle, Fumihisa Ono, Yoshihisa Mori, Naurang L. Saini, Pierre Cuq, Stephen Baghdiguian, Nelly Godefroy, Simon Galas

**Affiliations:** 1 IBMM, University of Montpellier, CNRS, ENSCM, Montpellier, France; 2 ISEM, University of Montpellier, CNRS, EPHE, IRD, Montpellier, France; 3 COMET, Platform Montpellier RIO Imaging, INM, University of Montpellier, INSERM, Montpellier, France; 4 Department of Applied Science, Okayama University of Science, Okayama, Japan; 5 Dipartimento di Fisica, Universita di Roma «La Sapienza», Roma, Italy

**Keywords:** Cellular imaging, Electron microscopy

## Abstract

Tardigrades can cope with adverse environmental conditions by turning into anhydrobiotes with a characteristic tun shape. Tun formation is an essential morphological adaptation for tardigrade entry into the anhydrobiotic state. The tun cell structure and ultrastructure have rarely been explored in tardigrades in general and never in *Hypsibius exemplaris*. We used transmission electron microscopy to compare cellular organization and ultrastructures between hydrated and anhydrobiotic *H. exemplaris*. Despite a globally similar cell organelle structure and a number of cells not significantly different between hydrated and desiccated tardigrades, reductions in the sizes of both cells and mitochondria were detected in dehydrated animals. Moreover, in anhydrobiotes, secretory active cells with a dense endoplasmic reticulum network were observed. Interestingly, these anhydrobiote-specific cells are in a close relationship with a specific extracellular structure surrounding each cell. It is possible that this rampart-like extracellular structure resulted from the accumulation of anhydrobiotic-specific material to protect the cells. Interestingly, after five hours of rehydration, the number of secretory cells decreased, and the specific extracellular structure began to disappear. Twenty-four hours after the beginning of rehydration, the cellular structure and ultrastructure were comparable to those observed in hydrated tardigrades.

## Introduction

Among all adaptative survival strategies that organisms have developed in response to harsh environmental conditions, cryptobiosis must be considered one of the most extreme. Tardigrades, which are invertebrates closely related to Arthropoda and Onychophora, are renowned for their remarkable cryptobiosis capabilities. According to Crowe’s^1^ study in 1975, four types of cryptobiosis are identifiable in tardigrades: (i) anhydrobiosis: induced by desiccation, (ii) cryobiosis: induced by freezing, (iii) anoxybiosis: induced by lack of oxygen and (iv) osmobiosis: induced by a high salt concentration. Among them, anhydrobiosis is considered the most common^1,2^.

While most animals have a limited ability to survive dehydration^3^, a few species in invertebrate taxa are able to survive complete water loss^4^. These organisms include bdelloid rotifers, nematodes and tardigrades, which have the ability to enter anhydrobiosis at any developmental stage^1,5–7^, and some dipteran larvae, such as those of the chironomid *Polypedilum vanderplanki*^8^. The process of anhydrobiosis is different depending on the animal. Indeed, when exposed to desiccation, the nematode *Ditylenchus dipsaci* coils into a tight spiral^9^, while tardigrades and rotifers form a tun to reduce their body surface area and rate of evaporation^10–12^. Nevertheless, little is known about how tardigrades survive such extreme stresses.

Numerous protein families and classes have been implicated in desiccation tolerance processes in other systems, including heat-shock proteins, antioxidant enzymes and some intrinsically disordered protein families^13–15^. For example, late embryogenesis abundant (LEA) proteins, belonging to the intrinsically disordered protein (IDP) family, have been found to stabilize membranes or sensitive enzymes during freezing or drying in a wide variety of organisms ranging from bacteria to plants and animals^13^. In plants, the LEA protein family vitrifies sugars to protect seeds and roots from drought^15^. The disaccharide trehalose has also been proposed to play a role in mediating desiccation tolerance. Nevertheless, although trehalose is essential for the protection of some organisms, such as *Caenorhabditis elegans*, *Saccharomyces cerevisiae* and chironomids, by vitrifying their cellular contents, other desiccation-tolerant animals, such as rotifers, do not require this sugar^16–19^. In Tardigrada, the presence and role of trehalose are still unclear. Some studies report low levels of this sugar, while others cannot identify trehalose at all in the same species^20–24^. It has not yet been studied in *Hypsibius exemplaris*.

Concerning morphological analyses, there are only a few reports on the ultrastructure of anhydrobiote animals^1,25–27^. Several studies on the ultrastructure of the hydrated tardigrade body wall have been conducted^28–33^, but studies on the ultrastructure of the tun stage are extremely rare. A study by Halberg *et al*.^34^ in 2013 described the tun morphology of the eutardigrade *Richtersius coronifer* with an emphasis on muscular organization. Studies by Czernekova *et al*.^35,36^ in 2017 and 2018 investigated the internal morphology of desiccated organs, tissues and cells in the same species. No reports have described the tun ultrastructure in *Hypsibius exemplaris*.

In the present study, we investigated the structure and ultrastructure of cells and organelles of anhydrobiotic *Hypsibius exemplaris* specimens by electron microscopy and compared them to the ultrastructure of active hydrated specimens. To characterize the process in greater detail, we enlarged our study to individuals rehydrated for 5 and 24 hours.

## Results

### *Comparison of hydrated and anhydrobiotic* Hypsibius exemplaris

#### Cell compaction

**Figure 1:**
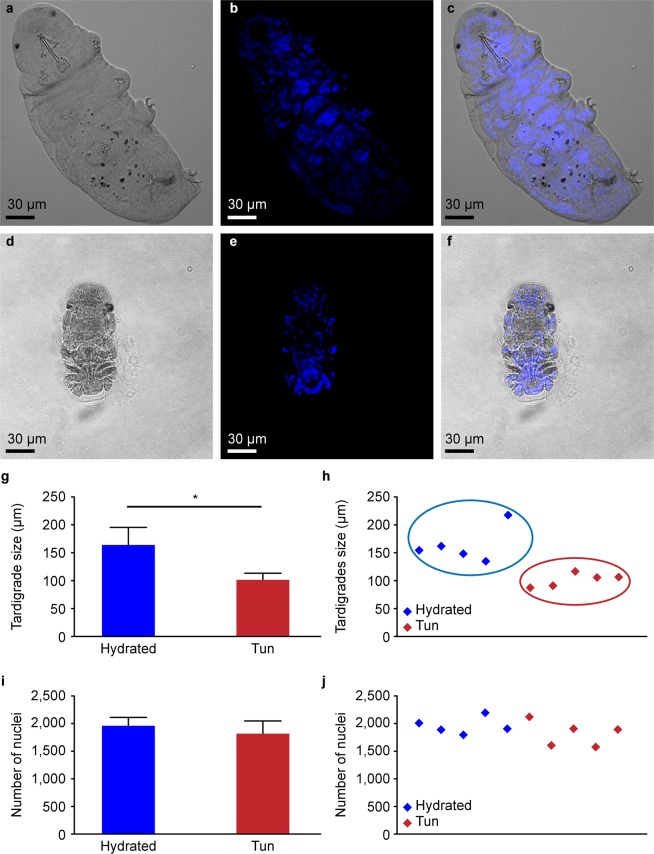
Comparison between hydrated and anhydrobiotic *H. exemplaris*: (**a**–**f**) Confocal microscopy images with DIC and DAPI staining. (**h**–**j**) Distribution of body sizes and nucleus numbers. (**g**–**i**) Statistics on body sizes and nucleus numbers. (**g**) Error bars indicate the standard deviation, and the star indicates a significant difference (Kolmogorov-Smirnov test, p = 0.036; α = 0.05). (**i**) Error bars indicate the standard deviation.

To compare the cell shapes and ultrastructures of anhydrobiotic and hydrated tardigrades, we dehydrated *Hypsibius exemplaris* for 6 days. The contraction of the body in the tun was observed by confocal microscopy with differential interference contrast (DIC) (Fig. 1a–f), revealing that hydrated tardigrades measured 164 +/− 32 µm, while tuns measured 101 +/− 12 µm (Fig. 1g). To clarify whether the compaction of the whole individual in the tuns group corresponded to cell compaction and not to a decreased global number of cells, we counted the DAPI-stained nuclei in both hydrated and dehydrated tardigrades. Experiments were performed on five individuals for each condition, and the distributions of nuclei numbers and body size are presented in Fig. 1h,j. A clear separation of the two populations of individuals was observed (Fig. 1h). The comparison of nuclei numbers (Fig. 1j) in hydrated individuals and tuns showed that despite a statistically significant decrease in size of approximately 38.4% between hydrated tardigrades and tuns, the number of cells did not significantly change (Fig. 1i,j).

#### Cell structure and ultrastructure

**Figure 2:**
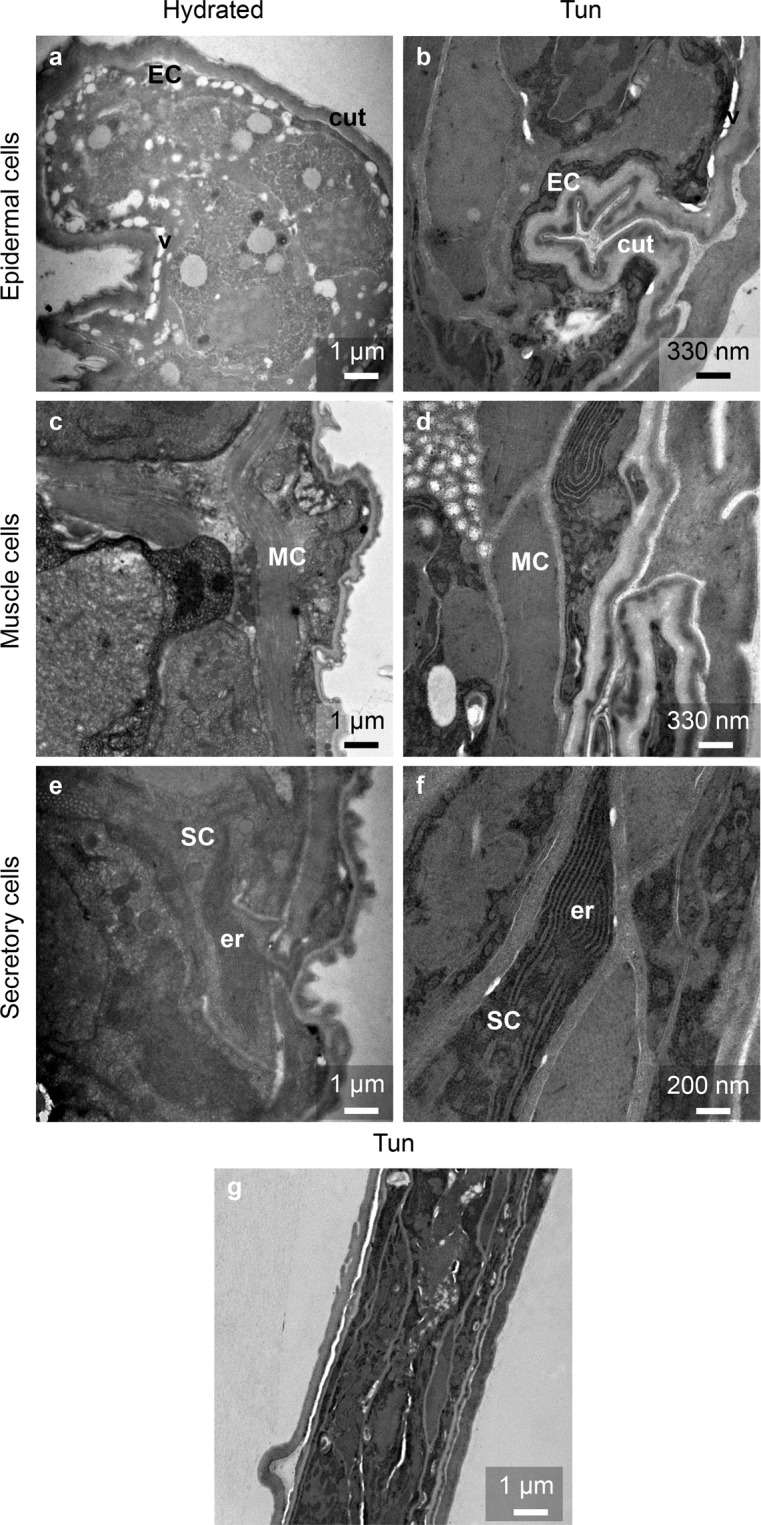
Ultrastructure of epidermal cells, muscle cells and secretory cells of *H. exemplaris* under both statuses: hydrated and tun. cut: cuticle, EC: epidermal cell, er: endoplasmic reticulum, MC: muscle cell, SC: secretory cell, v- vesicle.

**Figure 3:**
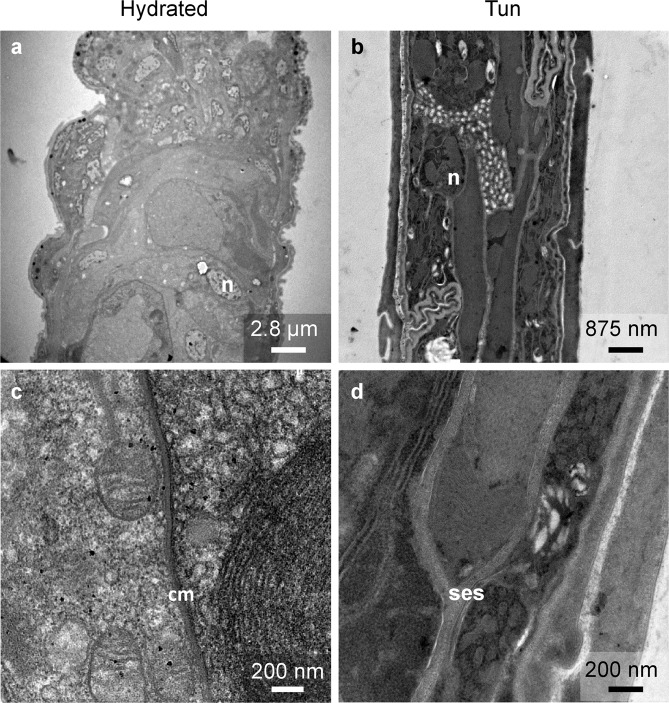
Comparison of extracellular structure between hydrated and anhydrobiotic *H. exemplaris*. cm: cell membrane, n: nucleus, ses: specific extracellular structure.

To compare the tissues of tuns and hydrated tardigrades, we performed a detailed transmission electron microscopy (TEM) analysis. Comparing the two types of individuals demonstrated that the cell compaction in tuns resulted in the presence of folds in the cuticle (Fig. 2b), which were not present in the hydrated tardigrades (Fig. 2a). Regarding the global structure of specific classically recognized cell types, the epidermal cells were comparable between the two groups, with an elongated morphology and numerous vesicles (Fig. 2a,b). Muscle cells in individuals of both physiological statuses possessed comparably long characteristic muscular fibers (Fig. 2c,d), while digestive cells exhibited the same microvillosity structures surrounding the gut (data not shown). On the other hand, tuns were shown to exhibit numerous secretory active cells, especially near muscle cells, with an abundant rough endoplasmic reticulum (Fig. 2f). These cells were rarely observed in hydrated individuals and contained substantially less abundant endoplasmic reticulum than tuns (Fig. 2e). Interestingly, the presence of these numerous secretory active cells correlated with the presence of a thick specific extracellular structure (SES) surrounding each cell in tun individuals (Fig. 3b,d). This structure was completely absent in hydrated tardigrade cells (Fig. 3a,c). Figure 2g presents an overview of a tun specimen at the same magnification as the hydrated individual for a better comparison of the global structure.

**Figure 4:**
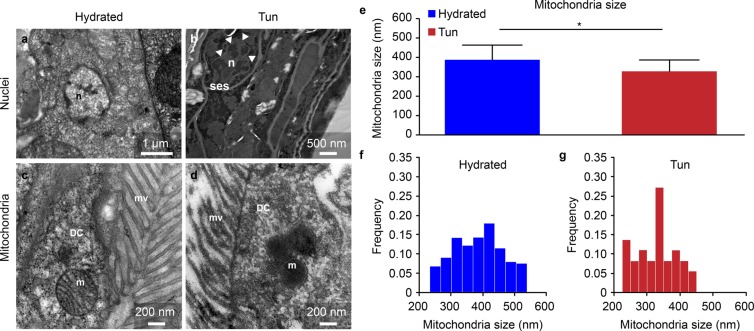
Comparison of nuclei and mitochondria between hydrated and anhydrobiotic *H. exemplaris*. (**a**–**d**) Transmission electron microscopy images. DC: digestive cell, m: mitochondria, mv: microvilli, n: nucleus, ses: specific extracellular structure. Arrowheads delimit the nuclear envelope. (**e**) Mean mitochondrial size. Error bars indicate the standard deviation, and the star indicates a significant difference (Student’s t-test, α = 0.05). Table 1 shows the complete statistical results. (**f**,**g**) Histogram of the mitochondrial size frequencies for hydrated tardigrades (F) and desiccated tardigrades (G).

In tardigrades of both statuses, hydrated and tuns, we observed comparable global organelle structures. Nuclei with euchromatin and heterochromatin were present in both hydrated and dehydrated tardigrade groups (Figs. 3a,b, 4a,b), and numerous mitochondria were also observed in both groups. The global mitochondrial structures were comparable regardless of the cell type (Fig. 4c,d and Supplementary Fig. S1), and the mitochondria were not degraded. The only difference was that the mitochondria of dehydrated *H. exemplaris* showed a statistically significant size reduction of 15% (Fig. 4e). Figure 4f,g show the size distributions of the 241 and 46 mitochondria measured in hydrated tardigrades and tuns, respectively. In parallel, the cristae in tuns appeared to be shorter than those in hydrated animals (Supplementary Fig. S1).

### *Study of* Hypsibius exemplaris *during rehydration*

To better understand the protection process observed in anhydrobiotic conditions, we studied individuals dehydrated for 6 days and rehydrated for 5 or 24 hours.

#### Tardigrades rehydrated for 5 hours

**Figure 5:**
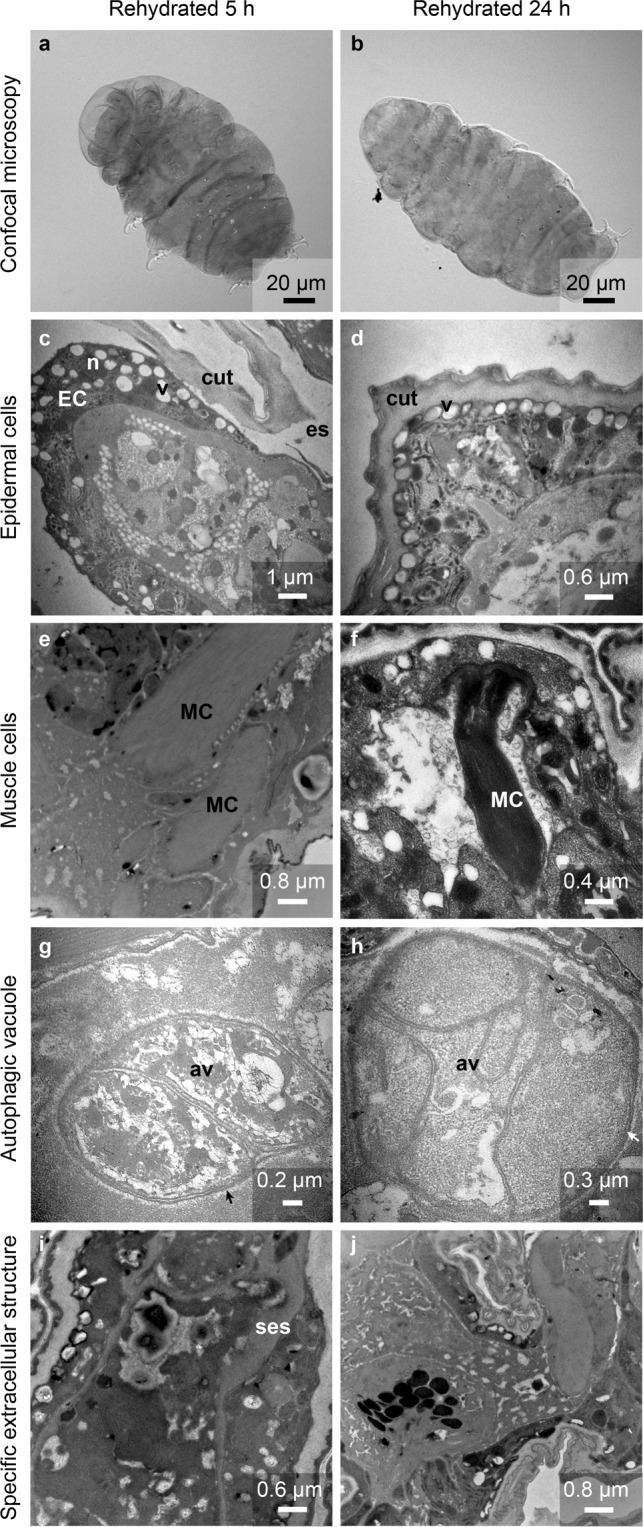
Description of *H. exemplaris* after 5 or 24 hours of rehydration. (**a**,**b**) Confocal microscopy images with DIC. (**c**,**d**) Ultrastructure of epidermal cells. (**e**,**f**) Ultrastructure of muscle cells. (**g**,**h**) Autophagic vacuole. (**i**,**j**) Specific extracellular structure. av: autophagic vacuole, cut: cuticle, EC: epidermal cell, es: extracellular space, MC: muscle cell, ses: specific extracellular structure. arrow: double membrane.

**Figure 6:**
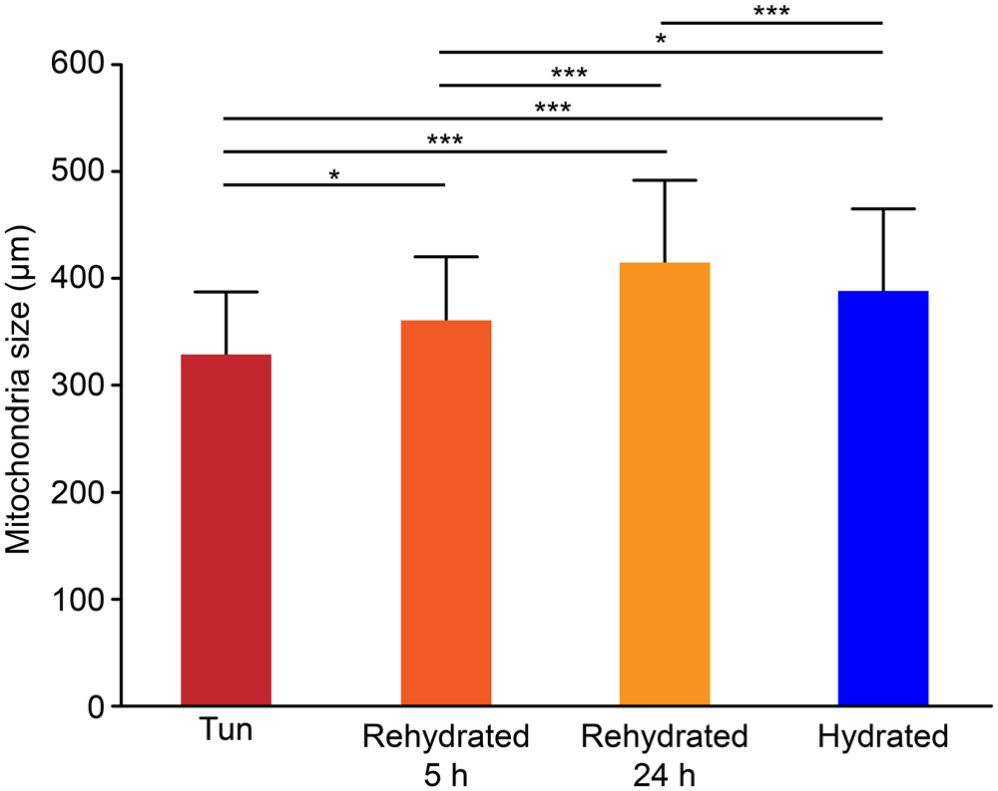
Mean mitochondrial sizes for four different types of *H. exemplaris*: tun specimens, specimens after 5 hours of rehydration, specimens after 24 hours of rehydration and hydrated specimens. Error bars indicate the standard error of the mean. <*> indicates a significant difference with p < 0.01 (Student’s t-test, α = 0.05). <***> indicates a significant difference with p < 0.0001 (Student’s t-test, α = 0.05). See Table 1 for the complete statistical results.

First, the animals in this condition were intermediate in size compared to those of hydrated and dehydrated individuals (Fig. 5a,b *versus* Fig. 1a,d). In correlation with these reduced sizes of cells and individuals, the epidermal cells are detached from the cuticle (Fig. 5c). This space observed between the cuticle and the cells was more important than in dehydrated tardigrades which possess folds in the cuticle that reduce the space (Fig. 2b). The global structure of epidermal cells with numerous vesicles was maintained (Fig. 5c *versus* Fig. 2a,b). Digestive cells also exhibited a classical structure comparable to that of both hydrated and dehydrated individuals (data not shown). Muscular cells exhibited a classical structure with long contractile fibers (Fig. 5e). Autophagic vesicles were observed at this stage (Fig. 5g). Interestingly, the number of secretory cells was decreased by rehydration, which was closely correlated with the disappearance of the SES (Fig. 5i). This disappearance was not regular or homogeneous because after 5 hours of rehydration, the thickness of the structure varied according to the cells. For some cells, the structure surrounding the cell was no longer detectable. Among organelles, the nuclei were comparable to those of hydrated tardigrades and tuns, and the size of the mitochondria was intermediate (Fig. 6). This observation was confirmed by statistical analysis (Table 1).

**Table 1:** Statistical results describing the mitochondrial sizes in the four different types of *H. exemplaris*: tun specimens (A), specimens after 5 hours of rehydration (B), specimens after 24 hours of rehydration (C) and hydrated specimens (D).

	Mean +/− SD	Code	Student’s t-test, α = 0.05	n=
Tun	327.94 +/− 58.72	A	p = 0.006 (B)	38
			p < 0.0001 (C)	
			p < 0.0001 (D)	
Rehydrated for 5 h	359.87 +/− 59.71	B	p = 0.006 (A)	89
			p < 0.0001 (C)	
			p = 0.003 (D)	
Rehydrated for 24 h	413.67 +/− 77.65	C	p < 0.0001 (A)	89
			p < 0.0001 (B)	
			p < 0.0001 (D)	
Hydrated	387.52 +/− 76.60	D	p < 0.0001 (A)	193
			p = 0.003 (B)	
			p < 0.0001 (C)	

#### Tardigrades rehydrated for 24 hours

At 24 hours after rehydration, the size of the tardigrades was already comparable to that of the hydrated tardigrades (Fig. 5b *versus* Fig. 1a). In agreement with this observation, epidermal cells were once again in close contact with the cuticle (Fig. 5d). Muscle cells exhibit classical long fibers like in other states (Fig. 5f *versus* 5e, 2c,d). Some autophagic vesicles were still present, but they contained less degraded material (Fig. 5h). We found no secretory cells in the five tested rehydrated individuals, which was in agreement with the total disappearance of the SES (Fig. 5j). Among organelles, the nuclei were comparable to those of hydrated tardigrades and tuns, and the mitochondria were larger than both the hydrated and tuns mitochondria (Fig. 6).

## Discussion

**Figure 7:**
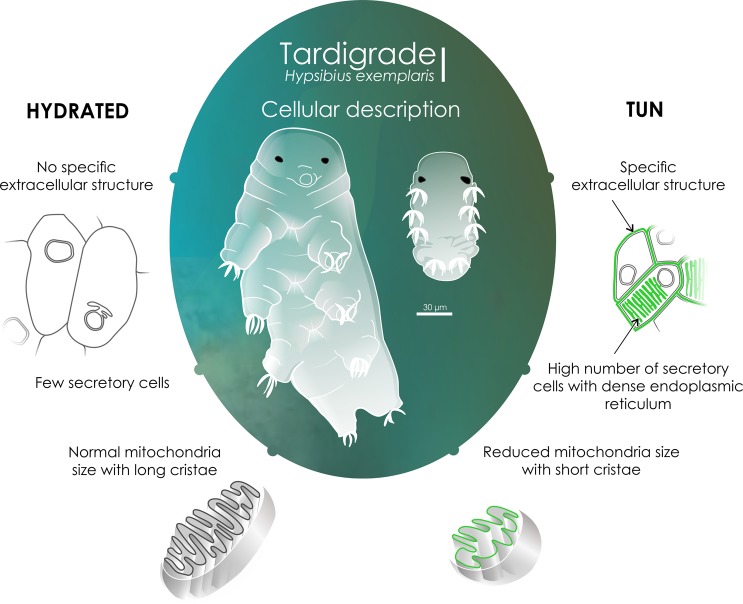
Cellular global description of *H. exemplaris* in tun and hydrated individuals. We thank Laurence Meslin from the platform “Communication Scientifique Visuelle” (ISEM, Montpellier, France) for the design of this figure.

Tardigrades are able to survive a wide variety of stresses, including desiccation. Understanding how they survive such extreme conditions is an important biological issue. Very few studies on this topic have been conducted, especially on *Hypsibius exemplaris*. Our results show that after six days of desiccation, the global structure and ultrastructure of the tissues are not greatly affected by the massive loss of water. This result is surprising given the importance of water for the structure of biological molecules and the amazing amount of water in normal cells. Not surprisingly, this loss of water results in a decrease of almost 40% in the size of individuals with accumulation of folds in the cuticle. This morphology is called tun. This size reduction corresponds to a reduction in the size of each of cell and not to a decrease in the number of cells. The water thus passes from the intracellular medium to the extracellular medium, possibly changing the intracellular salt concentrations. Nonetheless, most cells do not change their structure. Epidermal cells, digestive cells and muscle cells, three easily recognizable cell types, are comparable in the presence or absence of water. Note that the appearance of vesicles in epidermal cells as either electron-dense or electron-transparent was already described by Czernekova *et al*.^35^ in 2017. This phenomenon is explained by the fact that lipids are partly removed during fixation in some cases (independently to the hydration state). The differences that we observed are at the level of the numerous secretory cells that appear throughout the body in the tun (Fig. 7). These cells contain a very dense rough endoplasmic reticulum network, suggesting the existence of numerous secreted proteins in the tun that are not present in hydrated individuals. Interestingly, it was shown that tardigrades use specific IDPs (TDPs) to survive desiccation^37^. According to this study, TDP genes are constitutively expressed at high levels or induced during dehydration in many tardigrade species, including *H. exemplaris*. TDP expression levels are correlated directly with preconditioning (slow drying). Indeed, species requiring substantial preconditioning, such as *H. exemplaris* and *Paramacrobiotus richtersi*, exhibit upregulation of many TDPs induced by desiccation, while tardigrades that require little preconditioning, such as *M. tardigradum*, present constitutively high levels of TDPs. Among these TDPs, some are secreted and therefore produced by the rough endoplasmic reticulum, which is consistent with our results.

In parallel, we observed a thick SES (100 nm (+/−10)) surrounding all of the anhydrobiotic cells. It is possible that this extracellular structure results from the accumulation of anhydrobiotic-specific material such as TDPs, which are reported to form protective noncrystalline amorphous solids even in heterologous expression systems. Interestingly, the presence of this extracellular structure varied according to the rate of hydration since individuals rehydrated for 5 hours had an intermediate amount of this specific structure. These correlations were strong, as the number of secretory cells and the amount of rough endoplasmic reticulum they contained also decreased after 5 hours of rehydration. Twenty-four hours after rehydration, we did not observe any secretory cells, and the SES had completely disappeared in parallel to the increased cell and organism size. This result suggests that 24 hours of rehydration is sufficient for *H. exemplaris* to recover its physiological state. Note that some rare secretory cells might have remained, like in hydrated individuals, but we found none despite examining numerous sections due to the low numbers of occurrence. This return to the initial state upon hydration is in agreement with studies showing that disordered protein reorganization (formation of hydrogels, aggregation or vitrification) with stress is reversible (for review, see Chavali *et al*.^38^).

We have previously shown that the survival rate after dehydration is not 100% but rather close to 40%^39^. In agreement with this observation, some of the tuns we studied (not included in the five individuals selected for the study of tuns) lost their classical cellular structure. We think that these individuals corresponded to those who would never have woken up.

The last difference observed between hydrated and tun individuals was their mitochondria. Although their overall structures appeared quite normal, the mitochondria in tuns were smaller than those in hydrated individuals. This difference can be explained by the fact that mitochondria are dynamic and motile organelles that can fuse or split in physiological or toxic conditions^40–42^. The mitochondrial sizes are intimately linked to their activity and current surroundings. Moreover, compared to those in hydrated tardigrades, the mitochondria in tuns seemed to have shorter cristae, which were more difficult to discern with TEM. Because oxidative phosphorylation occurs mostly in the deeply invaginated cristae of the inner mitochondrial membrane^43–45^, we postulate that this decreased cristae size reflects the fact that the metabolism in tuns is minimal with limited breathing. In this way, the resumption of respiration with rehydration is accompanied by an increase in the mitochondrial size (visible after 5 hours rehydration). This increase must correspond to a gradual acceleration of metabolism, which could be made possible via autophagy. Indeed, we observed many autophagic vesicles in individuals at the beginning of the rehydration process. Even if we imagine that tardigrades protect themselves from the negative effects of oxidative phosphorylation by reducing their mitochondrial cristae, the simplest explanation is that autophagy, which is a highly conserved physiological process, allows tardigrades to resist nutritional starvation^46,47^. Since tardigrades do not eat when they are dehydrated, this process must provide the energy necessary for awakening and restoration of all biological processes, including muscle contraction. Moreover, autophagy allows the renewal of proteins and organelles damaged by stress conditions^48^. Individuals could therefore benefit from the renewal of molecules damaged by dehydration to recover energy.

We can conclude from our study that we observe the establishment of a rampart-like shaped extracellular structure in the tun that has never previously been described. Better characterizing this structure, which may contain TDPs produced by specific secretory cells, would be interesting to test its efficiency for heterologous expression systems.

## Methods

### Materials

In this study, we used *H. exemplaris* tardigrades (Sciento strain Z151), formerly known as *H. dujardini*^49^. This species resists dehydration and was the first to be sequenced^50–52^. Tardigrades were fed with the unicellular algae *Chlorococcum sp*. Both tardigrades and algae were purchased from Sciento Company (Manchester, UK). *H. exemplaris* were cultured in Petri dishes filled with Chalkley’s medium at 15 °C as previously described^53,54^.

### Desiccation protocol

To compare cell shape organization and ultrastructures between hydrated and anhydrobiotic *H. exemplaris*, tardigrade desiccation was performed. The desiccation protocol for adult tardigrades randomly selected from cultures was adapted from Hengherr *et al*.^22^. To facilitate later handling of the tardigrades, 20 specimens in a drop of water were placed on coverslips, which were then incubated at 25 °C in a sealed preconditioning box with 85% relative hygrometry (RH) for 16 hours with a saturated solution of KCl. The coverslips were then placed in a sealed box at 33% RH for 48 hours with an MgCl_2_-saturated solution. RH was controlled using a hygrometer. Full dehydration of the specimens was monitored by direct observation under a stereomicroscope. The anhydrobiotes were stored at the RH and temperature of the room for six days before analysis.

### Rehydration protocol

To rehydrate desiccated *Hypsibius exemplaris* after six days of dehydration, water droplets were added to the coverslips. Tardigrades were maintained in the medium at 15 °C and prepared for TEM after 5 and 24 hours in contact with liquid.

### Transmission electron microscopy

Samples were fixed in a solution of 2.5% glutaraldehyde in PHEM buffer (1×, pH 7.4) overnight at 4 °C. They were then rinsed in PHEM buffer and postfixed in 0.5% osmic acid for 2 hours in the dark at room temperature. After two rinses in PHEM buffer, the samples were dehydrated in a graded series of ethanol solutions (30–100%) and embedded in EmBed 812 using an automated microwave tissue processor for electronic microscopy (Leica EM AMW). Ultrathin sections (70 nm; Leica-Reichert Ultracut E) were collected at different levels of each block. These sections were counterstained with 1.5% uranyl acetate in 70% ethanol and lead citrate and observed using a Tecnai F20 transmission electron microscope at 200 kV at the CoMET MRI facilities (INM, Montpellier, France). For TEM, five tardigrades were analysed for each condition: tun, rehydrated for 5 hours, rehydrated for 24 hours and hydrated.

### Tardigrade size

We measured the sizes of hydrated and tun tardigrades. Animals were measured from the tip of the head to the extreme end of the body. Measurements from DIC images obtained using a Leica SPE confocal microscope at the DBS-Optique MRI facilities (Montpellier, France) were determined with ImageJ software. For each condition, five specimens were measured.

### Number of nuclei

We counted the number of nuclei in hydrated and tuned animals. Counts from DAPI z-stack stained images obtained using a Leica SPE confocal microscope at the DBS-Optique MRI facilities (Montpellier, France) were performed with ImageJ software. For each condition, the number of nuclei was counted in five tardigrades.

### Mitochondrial size

We measured the mitochondrial sizes in four conditions: hydrated animals, dehydrated animals, animals after 5 hours of rehydration and animals after 24 hours of rehydration. Measurements were performed with ImageJ software.

Between 46 and 241 mitochondrial size measurements were made for each condition as follows:

**Table:** 

*Condition*	*Number of measurements*
*Tun*	46
*Rehydrated for 5 hours*	109
*Rehydrated for 24 hours*	109
*Hydrated*	241

### Statistical analysis

We used XLSTAT software (Addinsoft, New York, NY, USA) to compare mitochondrial sizes among animals that were hydrated, rehydrated for 5 hours, rehydrated for 24 hours and dehydrated and to compare the body sizes and numbers of nuclei between hydrated and dehydrated animals.

## Supplementary information

Dataset 1.

## Data Availability

The datasets generated during the current study are available from the corresponding author upon reasonable request.
