# Supplementary material for: Ultrastructural analysis of the dehydrated tardigrade Hypsibius exemplaris unveils an anhydrobiotic-specific architecture
Source: Sci Rep. 2020 Mar 9;10:4324. doi: 10.1038/s41598-020-61165-1 (PMC7062702; doi:10.1038/s41598-020-61165-1)

**Ultrastructural analysis of the dehydrated tardigrade *Hypsibius exemplaris* unveils an anhydrobiotic-specific architecture**

Myriam Richaud, Emilie Le Goff, Chantal Cazevielle, Fumihisa Ono, Yoshihisa Mori, Naurang L. Saini, Pierre Cuq, Stephen Baghdiguian, Nelly Godefroy, Simon Galas

**Supplementary Fig. S1.** Comparison of mitochondria in epidermal, muscle and secretory cells between hydrated and anhydrobiotic *Hypsibius exemplaris*.

cut: cuticle, EP: epidermal cell, er: endoplasmic reticulum, m: mitochondria, MC: muscle cell, mf: muscle fibres, n: nucleus, SC: secretory cell.


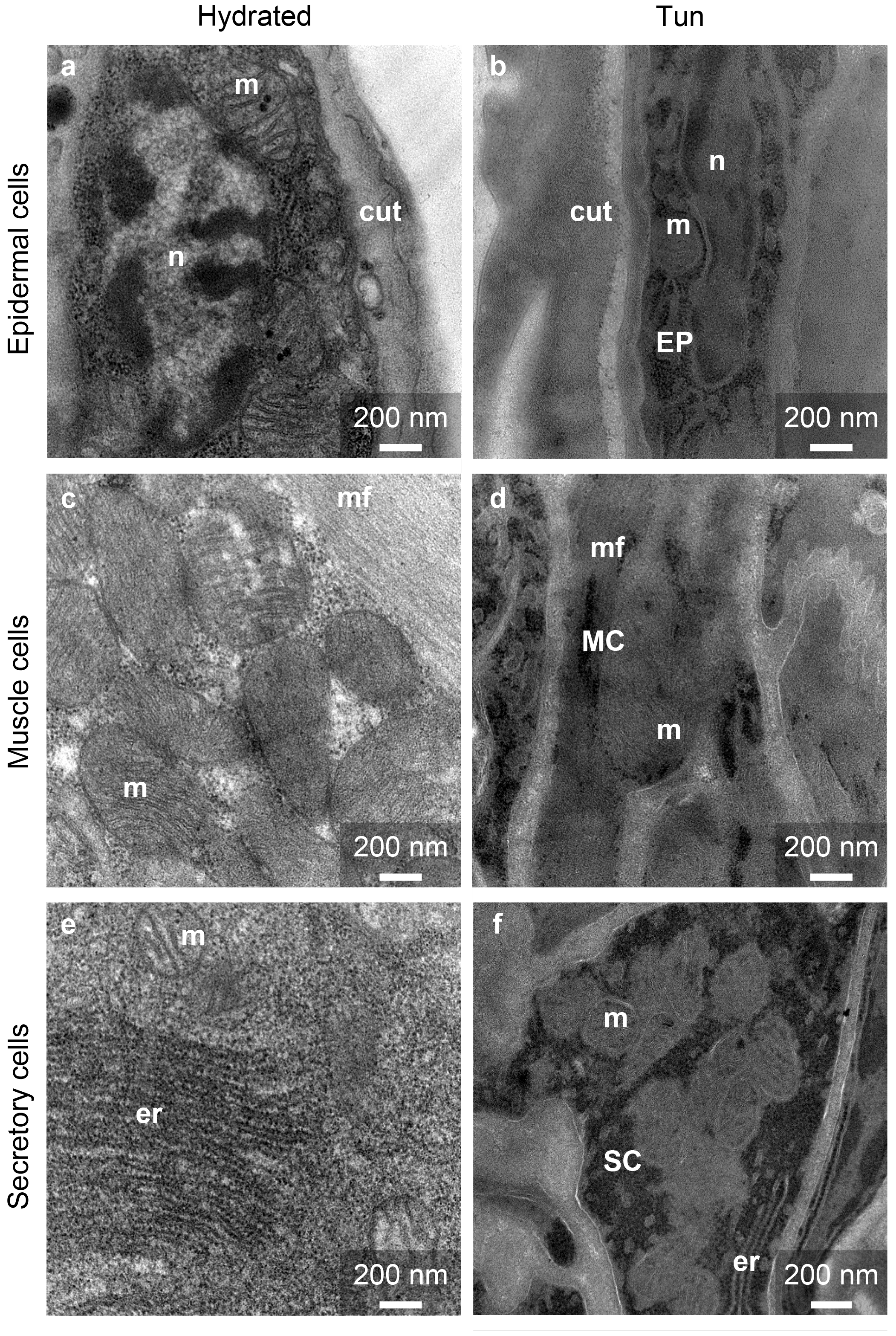

Supplement: Supplementary file 1 — Dataset 1. [file 41598_2020_61165_MOESM1_ESM.docx]
